# Inter‐organ communication: a gatekeeper for metabolic health

**DOI:** 10.15252/embr.201947903

**Published:** 2019-08-19

**Authors:** Judit Castillo‐Armengol, Lluis Fajas, Isabel C Lopez‐Mejia

**Affiliations:** ^1^ Center for Integrative Genomics University of Lausanne Lausanne Switzerland

**Keywords:** energy homeostasis, inter‐organ communication, metabolism, obesity, Metabolism

## Abstract

Multidirectional interactions between metabolic organs in the periphery and the central nervous system have evolved concomitantly with multicellular organisms to maintain whole‐body energy homeostasis and ensure the organism's adaptation to external cues. These interactions are altered in pathological conditions such as obesity and type 2 diabetes. Bioactive peptides and proteins, such as hormones and cytokines, produced by both peripheral organs and the central nervous system, are key messengers in this inter‐organ communication. Despite the early discovery of the first hormones more than 100 years ago, recent studies taking advantage of novel technologies have shed light on the multiple ways used by cells in the body to communicate and maintain energy balance. This review briefly summarizes well‐established concepts and focuses on recent advances describing how specific proteins and peptides mediate the crosstalk between gut, brain, and other peripheral metabolic organs in order to maintain energy homeostasis. Additionally, this review outlines how the improved knowledge about these inter‐organ networks is helping us to redefine therapeutic strategies in an effort to promote healthy living and fight metabolic disorders and other diseases.

**Glossary**
ACC: Acetyl‐CoA Carboxylase
ADBR3: β3‐adrenergic receptor
AdipoR1/2: Adiponectin receptors
AgRP: Agouti‐related peptide
AKT: Protein kinase B (PKB), also known as AKT
AMPK: AMP‐activated protein kinase.
Anorexigenic: Hormone(s) or compound(s) resulting in decreased appetite.
ARC: Arcuate nucleus
ATP: Adenosine trisphosphate
Autocrine signaling: signaling method in which a cell releases a signaling molecule that will bind to receptors and exert its action in the same cell.
BAs: Bile acids
BAT: Brown adipose tissue
BMP8b: Bone morphogenetic protein 8b
cAMP: Cyclic adenosine monophosphate
CCK: Cholecystokinin
CNS: Central nervous system
CSF: Cerebrospinal fluid
CTSB: Cathepsin B
CX43: Connexin 43
CXCL14: C‐X‐C motif chemokine ligand‐14
Cytokines: class of small proteins that can be produced by a broad variety of cells and act as signaling molecules. In circulation, cytokines are usually found in smaller concentration than hormones.
DIO2: Type 2 iodothyronine deiodinase
DIO: Diet‐induced obesity
DMH: Dorsomedial hypothalamus
Endocrine: signaling method in which a cell releases a signaling molecule that will be released into the bloodstream before binding to receptors and exerting its action in distant cells.
Exosomes: extracellular vesicles that are released from cells. Exosomes constitute a system for intercellular communication and for the transmission of macromolecules between cells. Exosomes may contain a broad variety of cargo molecules like proteins, lipids, DNA, mRNAs and miRNAs.
FBN1: Profibrillin gene. Profibrillin is the precursor of asprosin.
FGF19: Fibroblast growth factor 19
FGF21: Fibroblast growth factor 21
FNDC5: Fibronectin type III domain‐containing protein 5. FNDC5 is the precursor of irisin.
FoxO1: Forkhead box protein O1
GH: Growth hormone
GHSR: Growth hormone secretagogue receptor
GIP: Glucose‐dependent insulinotropic peptide
GLP‐1: Glucagon‐like peptide 1
GLP‐1R: GLP‐1 receptors
GLUT2: Glucose transporter 2
HFD: High‐fat diet
Hormones: class of proteins that act as signaling molecules. Hormones are secreted by a given tissue and are used to communicate between organs in order to trigger integrative responses to specific stimuli.
IL‐1Ra: Interleukin 1 receptor antagonist
IL‐6: Interleukine 6
IRS‐2: Insulin receptor substrate 2
JAK2: Janus kinase 2
Lcn5: Lipocalin 5
LEAP2: Liver‐expressed antimicrobial peptide 2
LepRb: Leptin receptor b
LHA: Lateral hypothalamic area
MAPK: Mitogen‐activating protein kinase
MCP1: Monocyte chemotactic protein 1
Metrnl: Meteorin‐like
mTOR: Mammalian target of rapamycin
NE: Norepinephrine
NPY: Neuropeptide Y
Orexigenic: Hormone(s) or compound(s) resulting in increased appetite.
Paracrine signaling: signaling method in which a cell releases a signaling molecule that will bind to receptors and exert its action in nearby cells.
PGC1α: Peroxisome proliferator‐activated receptor gamma coactivator 1‐α
PKA: Protein kinase A
PM20D1: Peptidase M20 domain‐containing 1
POA: Preoptic area
POMC: Pro‐opiomelanocortin
PSNS: Parasympathetic nervous system—The PSNS is part of the autonomic nervous system involved in the regulation of numerous basic body functions. It stimulates “feed and breed” and “rest and digest” functions and is antagonistic to the SNS.
RBP4: Retinol binding protein 4
RPA: Raphe pallidus
SctR: Secretin receptor
SNS: Sympathetic nervous system—The SNS is part of the autonomic nervous system involved in “fight or flight” responses. It stimulates rapid and urgent reactions and is antagonistic to the PSNS.
STAT3: Signal transducer and activator of transcription 3
T2D: Type 2 diabetes
T3, T4: Thyroid hormones
TCPTP: T‐cell protein tyrosine phosphatase
TGFβ: Transforming growth factor β
TGR5: BA receptor
TG: Triglycerides
TNF‐α: Tumor necrosis factor‐α
TSH: Thyroid‐stimulating hormone
UCP1: Uncoupling protein 1
VAN: Vagal afferent neurons
VMH: Ventromedial hypothalamus
WAT: White adipose tissue

## Introduction

In order to maintain homeostasis and adapt to external conditions, the different tissues of multicellular organisms communicate with each other via multiple signals. Peripheral organs produce a plethora of bioactive molecules, including hormones (from the Greek *horme* that means impulsion), that ensure intercellular signaling in an autocrine, paracrine, or endocrine manner (see Glossary). Peripheral organs and immune cells can also produce smaller bioactive proteins, namely cytokines 1, that also participate in inter‐organ communication. Alternatively, the nervous system coordinates whole‐body metabolism not only by the production of neurohormones that act locally, but also by direct innervation of the target tissues 2, 3, 4, 5. Indeed, sympathetic and parasympathetic fibers innervating peripheral tissues express enzymes crucial for the biosynthesis and transport of specific molecules (neurotransmitters and neuropeptides) necessary for the tissue‐specific response to external cues.

Early research from the 19^th^ century, most notably from Claude Bernard, first suggested that a system involving chemical messengers ensures the communication between the different organs of the body 6. The term “hormone” was first used in 1905 by the British physiologist Ernest Stalling to describe the gut hormone secretin, first described just 3 years prior 7, 8. Carl Ferdinand Cori and Gerty Cori then described the cycle in which lactate produced by anaerobic glycolysis in muscles can be recycled by the liver and converted to glucose. In turn, this glucose is returned to the muscle where it is metabolized to lactate 9. This “Cori cycle” was one of the first described examples of an efficient communication system between organs, which functions to facilitate the metabolic adaptation to energy demands.

Key metabolic hormones, like pancreatic insulin and glucagon, were successfully identified, synthesized, and used for therapy in the course of the 20^th^ century. The identification of signaling molecules produced by metabolic organs has increased exponentially in recent years, giving rise to the terms hepatokines 10, myokines 11, adipokines 12, and batokines 13, to describe the hormones produced by liver, muscle, white adipose tissue (WAT), and brown adipose tissue (BAT), respectively. The secretion of these signaling molecules varies according to the metabolic status of the body. They respond for instance to fasting and feeding cycles 14, to the circadian rhythm 15, to cold exposure 16, and to exercise^17^, thus participating in the organism's adaptive response and ensuring metabolic flexibility. Inter‐organ communication is altered in different pathologic conditions, for example, in conditions related to adipose tissue dysfunction, like obesity. As such, alterations in hormones and cytokines are currently known to notably contribute to the spectrum of obesity‐associated pathologies. Therefore, pharmacological interventions to modify the production of hormones/cytokines, or directly delivering recombinant hormones/cytokines, are currently being explored as promising approaches to treat a wide variety of obesity‐related endocrine diseases. In this review, we summarize well‐established mechanisms of inter‐organ communication and focus on how recent research has highlighted the importance of the crosstalk between gut, brain, and other specific peripheral metabolic organs, namely liver, muscle, WAT, BAT, and pancreas, in the maintenance of metabolic fitness. The role of the immune system in energy homeostasis has been recently discussed elsewhere 18 and is not discussed in this review. This review further concludes by citing additional actors and mediators of inter‐organ communication and states our opinion about the future directions of the field.

## Inter‐organ communication in the control of fasting/feeding cycles

**Figure 1:**
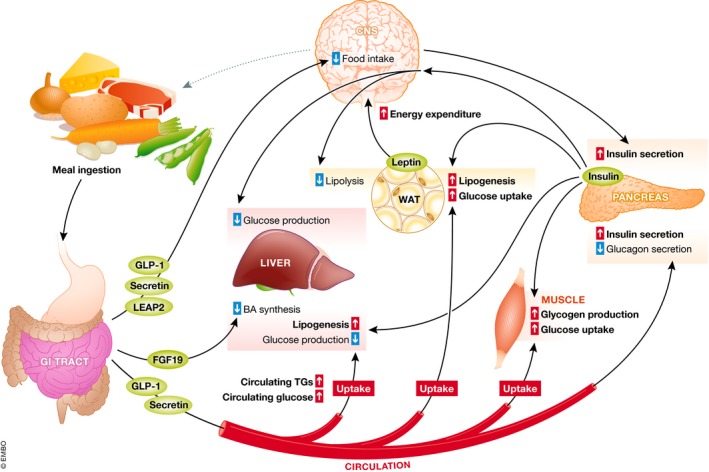
Inter‐organ communication under feeding conditions Food ingestion stimulates the secretion of several molecules such as GLP‐1, secretin and LEAP2. These gut hormones signal to the brain to reduce food intake. FGF19 is produced by the intestine and reduces bile acid (BA) synthesis. GLP‐1 and secretin will also stimulate insulin (and reduce glucagon) secretion by the pancreas. In turn, insulin will promote glycogen production and glucose uptake in muscle, decrease glucose production and increase lipogenesis in liver, and increase glucose uptake and lipogenesis from circulating glucose and triglycerides (TGs) in WAT. Leptin, produced by white adipocytes, will act in the CNS to repress food intake. Moreover, insulin can target the brain in order to decrease lipolysis in WAT and glucose production in liver.

**Figure 2:**
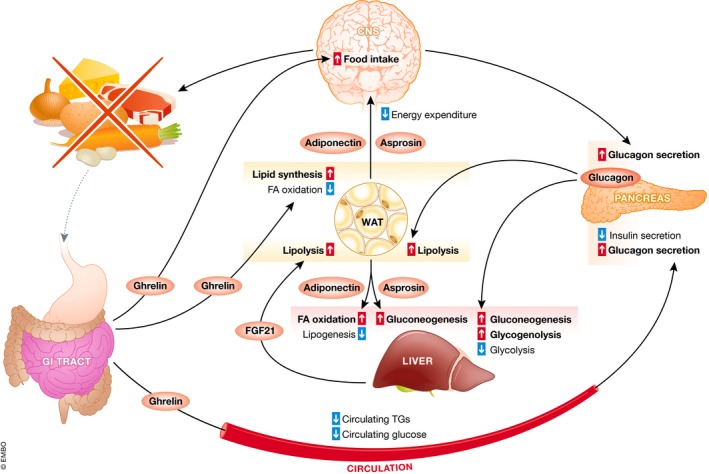
Inter‐organ communication under fasting conditions Ghrelin is a gut hormone secreted under fasting conditions. It targets the brain to increase food intake. Pancreatic glucagon secretion is also increased by ghrelin, and directly by low blood glucose levels. Glucagon will target the liver to decrease glycolysis and increase hepatic gluconeogenesis and glycogenolysis, as well as WAT to increase lipolysis. Ghrelin can promote adiposity by increasing WAT lipid synthesis and reducing WAT fatty acid (FA) oxidation. Fasting also stimulates the secretion of the adipokines asprosin and adiponectin that will act through the brain to decrease energy expenditure and promote food intake. Asprosin can also increase hepatic gluconeogenesis, whereas adiponectin reduces lipogenesis and increases hepatic FA oxidation. Increased hepatic FGF21 levels under fasting conditions increase lipolysis in WAT.

In this section, we will first discuss the organismal adaptation to the consumption of food (Fig 1), followed by the compensatory response engaged in energy low conditions like the fasting state (Fig 2).

### Inter‐organ communication in feeding conditions

The physiological response to a meal ingestion initiates in the gut and is triggered by the presence of nutrients. Enteroendocrine cells sense nutrients in the intestinal lumen and produce peptides such as cholecystokinin (CCK), secretin, and the incretins; glucose‐dependent insulinotropic peptide (GIP); and glucagon‐like peptide 1 (GLP‐1) 19, 20. Gut hormones play their role by directly acting on target tissues via the circulation or by activating intestinal neurons in a paracrine manner. A major target for gut‐derived hormones is the vagal afferent neurons (VANs) 21. Endogenous GLP‐1 acts on VANs to inhibit food intake. The actions of GLP‐1 are further modulated by the fact that GLP‐1 receptors (GLP‐1R) are internalized upon fasting conditions, but translocate to the plasma membrane after a meal when ghrelin levels are low. GLP‐1 and GIP work synergistically to potentiate glucose‐stimulated insulin secretion by pancreatic β‐cells 21. Moreover, GLP‐1 inhibits glucagon production by pancreatic α‐cells. Secretin stimulates exocrine pancreas activity to ensure a proper environment for digestion and absorption of nutrients. Moreover, secretin can also inhibit food intake via the activation of secretin receptor (SctR) in vagal sensory nerves and melanocortin signaling in the brain 22, 23.

After the postprandial regulation initiated in the gut, changes in circulating levels of nutrients, mainly glucose and triglycerides (TG), also engage the response of several peripheral organs and the nervous system. An increase in circulating glucose levels is sensed by pancreatic β‐cells through the glucose transporter GLUT2. Glucose sensing leads to a β‐cell response that ultimately leads to insulin secretion 24. In parallel, glucose inhibits the release of glucagon from the pancreatic α‐cells 25, 26. Insulin targets insulin‐sensitive organs, like the liver, muscle, WAT, and brain to induce glucose utilization and storage thus preventing hyperglycemia. By signaling through the brain, insulin regulates feeding behavior and modifies energy metabolism in liver and adipose tissues, as well as several more cognitive functions 27, 28, 29, 30, 31. In the liver, insulin promotes metabolization of glucose by hexokinases and *de novo* lipogenesis, while decreasing hepatic glucose production. Intact hepatic insulin signaling is, however, dispensable for the postprandial repression of hepatic glucose production in the absence of hepatic FoxO1 32, 33, highlighting the importance of hypothalamic insulin signaling in the control of liver glucose metabolism 34. Insulin also triggers glucose uptake by promoting the externalization of the glucose transporter GLUT4 in WAT and muscle, glycogen synthesis in muscle, and *de novo* lipogenesis by promoting the expression and activity of fatty acid synthesis enzymes specifically in WAT. In WAT, glucose‐derived glycerol serves as building blocks for TGs; in turn, TGs serve as a fuel stock for conditions of energy scarcity. In order to avoid excessive food intake, excessive adiposity, and unnecessary energy storage, which have deleterious effects for the organism, WAT also has the capability to act as a hormone‐producing organ. Leptin is secreted by white adipocytes proportionally to TG stores 35, 36, 37. Leptin is widely known to target the central nervous system that highly expresses the long form of leptin receptor (LepRb). By binding LepRb and signaling via JAK2 and STAT3 in the brain, leptin signals to maintain a constant energy stock. Indeed, leptin suppresses food intake and the production of adrenal corticosteroids while promoting energy expenditure and decreasing insulin secretion 38, 39. Leptin can also activate glucose uptake in BAT, as well as glucose uptake and fatty acid oxidation in muscle in an AMPK‐dependent manner 40. Whether this process is direct or indirect remains unclear 41.

In feeding conditions, the bile acids (BAs) released into the intestinal lumen also induce the expression of FGF19 (Fgf15 in mice) in the ileum. FGF19 then signals to the liver and induces a feedback repression of hepatic BA synthesis 42. FGF19 also increases liver protein and glycogen synthesis while repressing hepatic glucose production 43, 44, in order to ensure proper postprandial energy storage independently from insulin. Importantly, Fgf15 can also signal through the brain to modulate glucose homeostasis 45 and glucagon secretion 46.

More recently, the liver‐expressed antimicrobial peptide 2 (LEAP2), a peptide initially described as an antimicrobial liver‐produced peptide, was also described to be a gut‐derived peptide. LEAP2 is produced by the liver and the small intestine in the fed state, and its secretion is suppressed by fasting. Importantly, LEAP2 was reported to be an endogenous antagonist of the ghrelin receptor, or growth hormone secretagogue receptor (GHSR). Indeed, LEAP2 can fully blunt GHSR activation by ghrelin, thus reducing food intake and growth hormone (GH) release 47. This recent study adds LEAP2 to the growing list of hormones that connect gut, brain, and other peripheral organs in metabolic control.

Thus far, we have described how several organs from the body, mainly gut, brain, pancreas, and WAT communicate in the fed state. Upon ingestion of a meal, the aforementioned organs orchestrate a coordinated response in order to ensure proper digestion and storage of energy substrates, while initiating a negative feedback loop involving satiety and increased energy expenditure to maintain energy homeostasis by preventing an excessive “positive energy balance” (Fig 1).

### Inter‐organ communication in fasting conditions

When the organism's energy levels are low, the lack of food in the stomach, as well as the decrease in blood glucose, are sensed in order to replenish energy stores and to produce alternative substrates to keep organs functioning. Ghrelin is mainly secreted by the gastric epithelium when the stomach is empty. Plasma concentrations of ghrelin are high during fasting and decrease in the postprandial state 48. Ghrelin was first described as a potent inducer of GH secretion 49, but it is now known to stimulate food intake and adiposity and to maintain glucose levels by binding to its receptor GHSR. Ghrelin exerts its orexigenic effects centrally in the arcuate nucleus (ARC) of the hypothalamus by triggering the expression of agouti‐related protein (AgRP) and neuropeptide Y (NPY) in order to increase appetite. However, the exact mechanism by which ghrelin plays its central role remains unclear. To date, three hypotheses have been suggested as follows: (i) Ghrelin acts by activating VANs, (ii) ghrelin is synthesized locally in response to feeding, and (iii) ghrelin crosses the blood–brain barrier and activates its receptor in the hypothalamus 50. Ghrelin attenuates insulin production and increases blood glucose concentration by promoting the intracellular localization of the pancreatic β‐cell GLP‐1R under fasting conditions, thus counteracting GLP‐1 signaling 21. Ghrelin also stimulates glucagon secretion in pancreatic α‐cells 51. Moreover, ghrelin can directly promote adiposity, independently of food intake or GH secretion, by increasing carbohydrate use, stimulating lipid synthesis, and reducing fatty acid oxidation 50, 52. The effects of ghrelin on adiposity have been shown to require intact sympathetic nervous system signaling 53.

Adipokine secretion is also modified in low energy conditions. Leptin circulating levels rapidly decrease upon fasting. This reduction in leptin promotes a switch between the “fasted state” and the “fed state” by promoting food intake and reducing energy expenditure 54. In contrast to leptin, adiponectin levels increase in low glucose and low insulin conditions, at least in the cerebrospinal fluid (CSF) 55, 56. Overall, adiponectin levels are inversely proportional to adiposity and correlate with improved metabolic fitness. Numerous studies have reported that adiponectin targets numerous tissues (adiponectin receptors AdipoR1/2 are essentially ubiquitous). In the brain, adiponectin activates AMPK signaling in hypothalamic ARC, resulting in an increase in food intake and a reduction in energy expenditure, suggesting that in the hypothalamus adiponectin promotes feeding and inhibits the anorexigenic effects of leptin 55, 56. In the peripheral tissues, adiponectin promotes liver insulin sensitivity via several independent mechanisms: an AMPK‐dependent mechanism involving increased ACC phosphorylation, an AMPK‐independent mechanism that involves a reduction in liver ceramide levels 57, and an IL‐6‐dependent mechanism that involves the upregulation of insulin receptor substrate 2 (IRS‐2) through the activation of STAT3 58. Adiponectin also reduces hepatic *de novo* lipid synthesis and increases hepatic fatty acid oxidation 39 but its effects in hepatic glucose production remain debated 39.

Asprosin, a novel fasting‐induced adipokine that promotes hepatic glucose production, was recently identified 59. Asprosin is the C‐terminal cleavage product of profibrillin (*FBN1* gene). It is encoded by the last two exons of *FBN1* and is mainly produced by adipose tissue in mice. Asprosin directly targets the liver to promote glucose release, without increasing plasma levels of glucagon, catecholamines, and glucocorticoids. This process is independent from glucagon receptor signaling and from the β‐adrenergic receptor 59. Additionally, asprosin can directly target the central nervous system (CNS) to increase food intake 60.

Fibroblast growth factor 21 (FGF21) was first identified as an hepatokine 61 and has been shown to be necessary for the adaptation to fasting 62, 63. Indeed, liver expression and plasma levels of FGF21 are increased upon fasting. FGF21 in turn increases adipose tissue lipolysis, as well as hepatic fatty acid oxidation and ketogenesis, thus ensuring the availability of substrates for the brain. It is worth noting that FGF21 knockout mice exhibit lower glycemia in the fasted state, and lower plasma levels of ketone bodies in both the fasted and the fed state 64, 65.

Fasting‐induced hypoglycemia also results in the stimulated secretion of glucagon. Hypoglycemia is directly sensed by GLUT2‐positive hypothalamic neurons that promote glucagon secretion via an increase in parasympathetic input 66. Glucagon signals to the liver through the PKA signaling pathway to stimulate (i) the mobilization of hepatic glycogen (glycogenolysis) and, if the fasting is prolonged, (ii) hepatic *de novo* glucose synthesis (gluconeogenesis), and (iii) to repress glycolysis and glycogenesis. To ensure the availability of alternative substrates for the peripheral organs, and to preserve glucose production for the brain, glucagon also triggers lipolysis in WAT. TGs are metabolized into free fatty acids and glycerol. Glycerol is transported to the liver where it is oxidized or used as a substrate for gluconeogenesis. Albumin‐bound fatty acids are transported in the bloodstream to serve as oxidation substrates for the liver, muscle, and other tissues. WAT lipolysis can also directly be promoted by sympathetic nervous system (SNS) innervation. Indeed, SNS fibers can release norepinephrine (NE) locally and exert similar effects to glucagon. This increased SNS tone in WAT could be directly triggered by hypoglycemia sensing in the brain 67, 68. Notably, direct innervation of WAT depots is sparser than in BAT 69. However, it is denser in specific regions of subcutaneous (or inguinal) WAT and can be increased in response to cold 70, 71. Therefore, cell‐to‐cell communication via connexin 43 (CX43) containing gap junctions is essential to disseminating adrenergic activation signals 72.

In this section, we described inter‐organ communication in situations of food deprivation (Fig 2). This counter regulatory response promotes food intake, maintains blood glucose levels high enough to prevent alterations in brain function, and ensures that energy‐providing substrates are available to all organs. This is accompanied by an overall reduction of energy expenditure in order to preserve energy.

## Inter‐organ communication in non‐shivering thermogenesis

**Figure 3:**
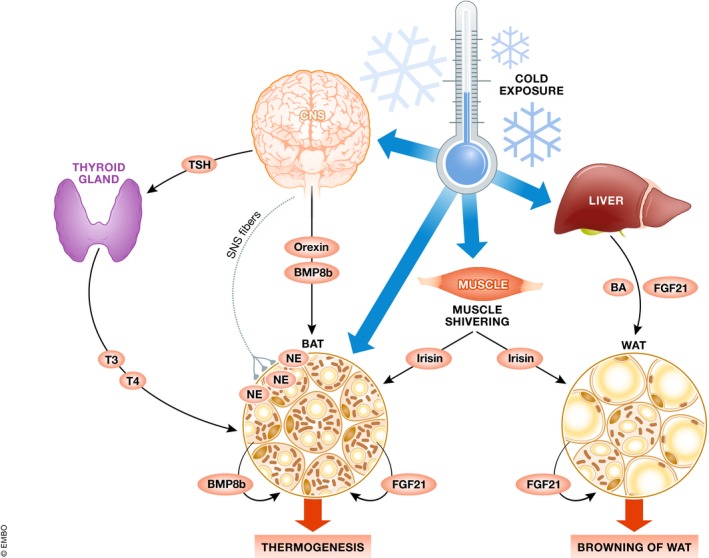
Inter‐organ communication under cold exposure Cold stimuli, sensed by neurons in the skin, activate the thermoregulatory hypothalamic regions of the brain that will secrete orexin and BMP8B to stimulate BAT thermogenesis. Moreover, the SNS is responsible for the local production of NE in BAT. The hypothalamic–pituitary–thyroid axis is also activated in response to cold and promotes the release of thyroid hormones (T3 and T4) in order to contribute to the activation of BAT thermogenesis. Cold exposure can also trigger muscle shivering and thus the production of the myokine irisin. Irisin can in turn stimulate BAT thermogenesis. If cold exposure is sustained over time, WAT undergoes browning and can contribute to thermogenesis. Irisin is one of the molecules that can trigger this process. In addition, cold‐dependent hepatic FGF21 and BA secretion contribute to the browning of WAT. Finally, FGF21 can be produced by WAT; and BMP8b and FGF21 can be produced by BAT, after cold activation and promote thermogenesis in a paracrine manner.

In order to adapt to cold exposure and maintain body temperature, different strategies have been developed by homeotherms. Mammals have BAT, a highly specialized tissue that functions to produce heat 73. BAT is especially abundant in hibernating mammals 74 *,* but can also be found in adult humans 75. Inter‐organ communication in response to cold stress is summarized in Fig 3.

### Cold‐induced thermogenesis

The main organ that integrates the response to cold is the CNS. Cold is sensed by the terminals of sensory neurons found in skin, and this signal is then processed in the hypothalamic preoptic area (POA), also known as the thermoregulatory center 76. The transduction of the signal will stimulate other hypothalamic regions, namely the lateral hypothalamic area (LHA), the ventromedial hypothalamus (VMH), the dorsomedial hypothalamus (DMH), and the arcuate nucleus (ARC).

Orexin producing neurons that are located in the LHA, named orexin neurons, participate in the regulation of thermogenesis. The central administration of orexin peptide has been described to stimulate BAT activation and therefore thermogenesis 77. Moreover, orexin infusion into the VMH can stimulate the sympathetic firing in BAT 78. Both the LHA and the VMH can synergistically interact and activate BAT. Hypothalamic expression of bone morphogenetic protein 8b (BMP8b) in the VMH stimulates orexin secretion in the LHA through a decrease in AMPK activity and thus enhances the firing of projections that will stimulate BAT thermogenesis 79. Interestingly, BMP8b can also be produced in BAT and promote NE signaling locally, activating the MAPK pathway and lipolysis in brown adipocytes 80.

Several studies have linked the DMH with the response to cold. For example, a chemical stimulation of this area increases the thermogenic response in rats 81. Similarly, administration of the incretin hormone GLP‐1 in the DMH increases BAT thermogenesis 82. Outflow from these areas is triggered by the premotor neurons found in the brain stem region raphe pallidus (RPA). It has been broadly discussed that sympathetic nerves are present within the parenchyma of adipose tissue 83. These nerves express tyrosine hydroxylase, the enzyme responsible for the secretion of the catecholamine hormone NE 84. BAT is a major catecholamine‐responsive tissue. It is mainly constituted by brown adipocytes that contain numerous lipid droplets and a high number of mitochondria. NE stimulates the β3‐adrenergic receptor (ADBR3) and activates the cAMP/PKA pathway, thus triggering lipolysis and increasing the expression of uncoupling protein 1 (UCP1). Free fatty acids, resulting from lipolysis, are used both as energy substrates and as activators of UCP1 to uncouple ATP production from mitochondrial respiration into heat 73, 85.

Apart from catecholamines, thyroid hormones can also trigger BAT activity 86. The thyroid hormones T3 and T4 are synthetized in the thyroid gland upon hypothalamic stimulation of the pituitary gland and thyroid‐stimulating hormone (TSH) production 87. Thyroid hormones access brown adipocytes from the bloodstream through specific transporters. In brown adipocytes, T4 can be converted to its active form T3 via type 2 iodothyronine deiodinase (DIO2) thus compensating for the low levels of thyroid gland‐secreted T3 88. T3 directly activates lipolysis through the PKA pathway. The relevance of thyroid hormone regulation for BAT function is illustrated by the blunted thermogenic response observed in DIO2 knockout mice, despite their increased UCP1 expression 89.

If cold stress is sustained over time, a process known as browning is triggered. This process is characterized by the appearance of brite (from *br*own‐in‐wh*ite*) adipocytes, also called beige adipocytes, mainly in subcutaneous WAT. Brite adipocytes can express UCP1 and, as such, can participate in the maintenance of body temperature 90. UCP1‐expressing adipocytes (brown and brite) are also capable of participating in the regulation of glucose homeostasis due to their high capability to uptake glucose. Moreover, they can contribute significantly to the clearance of circulating lipids, increasing insulin sensitivity. Their potential contribution to global energy expenditure has opened the door to new human therapeutic strategies against obesity and diabetes.

Under cold exposure, FGF21 can be secreted by BAT and WAT, have a paracrine effect, and stimulate the expression of thermogenic genes 91. Some teams have reported that FGF21 knockout mice have impaired adaptation to chronic cold exposure and decreased browning of WAT 92. However, others have shown that the lack of FGF21 in long‐term cold adaptation does not impair the thermogenic response of mice 93. Despite the evidence of FGF21 production in WAT, controversial results were published by Véniant *et al* 94 who demonstrated that beneficial effects of weight loss observed after cold were not directly related to FGF21‐induced browning. Additionally, hepatic FGF21 production can also be induced by cold, and blunted hepatic FGF21 secretion, but not adipocytic FGF21 secretion, results in impaired cold tolerance and decreased sympathetic nerve activity in BAT 95. Therefore, further studies are needed to elucidate the role of FGF21 in the control of non‐shivering thermogenesis.

Besides their role in the digestion of dietary lipids, hepatic BAs can be induced by cold exposure 96. It has been described that BA secretion increases energy expenditure in BAT by enhancing the enzymatic activity of DIO2 and therefore regulating thyroid hormone metabolism in BAT 97. Moreover, studies using BA receptor TGR5 adipose tissue‐specific knockout mice proved that BAs also participate in the browning of WAT. These mice show neglectable browning and exhibit reduced cold tolerance due to reduced adipose mitochondrial fusion 98.

Historically, UCP1 has been considered indispensable for thermogenesis in BAT. However, recent research demonstrates that UCP1 is not essential for non‐shivering thermogenesis 93, 99, 100. Peptidase M20 domain‐containing 1 (PM20D1), a secreted enzyme produced by brown and brite adipocytes, has been recently discovered to contribute to this alternative thermogenesis by participating in the uncoupling of mitochondria in an UCP1‐independent manner 99, 100.

### Feeding‐induced thermogenesis

Cold is not the only stimulus that activates non‐shivering thermogenesis. It has been demonstrated by several groups that there is a meal‐associated thermogenesis 101, 102, 103. The activation of feeding–responsive thermogenesis has been strongly associated with food intake regulation by inhibiting orexigenic activation in the brain, revealing a gut–brain–BAT–brain axis 104. In particular, feeding activates the secretion of periprandial gut hormones, such as CCK, GLP‐1, or secretin 105, 106, 107. Most of these molecules act through the brain, like GLP‐1 and CCK, where they increase sympathetic activation and therefore stimulate the local release of NE in BAT. Interestingly, secretin can stimulate BAT thermogenesis in a non‐sympathetic manner and contribute to satiation. A secretin injection in fasted mice directly activates BAT thermogenesis by increasing lipolysis through the cAMP‐PKA pathway. Moreover, secretin treatment increases the expression of the anorexigenic proopiomelanocortin (POMC) peptide in the hypothalamus and therefore reduces food intake 107.

Apart from its role in the regulation of food intake described above, leptin can increase energy expenditure by inducing BAT activation. The actions of leptin and insulin in POMC neurons can also be triggered by a decrease in the hypothalamic levels of T‐cell protein tyrosine phosphatase (TCPTP), which is a negative regulator of insulin signaling, to further increase the browning of WAT and energy expenditure 108, 109. This mechanism can limit the effects of diet‐induced obesity (DIO).

In this section, we described how brown and beige adipose tissue thermogenesis are activated not only to adapt to cold stress (Fig 3), but also to prevent the excessive energy storage that could lead to obesity and thus maintain general energy homeostasis in the organism.

## Inter‐organ communication during exercise

Physical exercise was first described to have beneficial effects for health in 450 BC by Hippocrates 110. Despite early hypotheses suggesting that muscles release a hypoglycemic “humoral factor” in response to glucose demand 111, part of the molecular mechanisms behind this observation have only been described rather recently. Indeed, physical exercise is not only beneficial because of the immediate increase in energy expenditure; it also remodels whole‐body energy metabolism. The ensemble of muscle‐derived peptides and proteins, currently referred to as myokines, is at least in part, responsible for exercise whole‐body health benefits. Recent analyses of muscle secretomes revealed that aerobic exercise or strength training trigger the secretion of numerous myokines 11. Importantly, it is worth noting that the production of “positive” myokines is as much promoted by physical exercise as it is repressed by physical inactivity, highlighting the importance of lifestyle for healthspan 112.

More precisely, during exercise skeletal muscle tightly interacts with adipose tissue, pancreas, and liver to sustain the energy demands of physical activity and mediate positive effects in whole‐body energy metabolism. The first exercise‐induced myokine to be described was interleukin 6 (IL‐6) 113. Exercise‐induced IL‐6 production was initially believed to be linked to muscle damage, but has now been proven to directly depend on contraction and to mediate muscle glucose uptake, increased insulin sensitivity, and increased fatty acid oxidation. IL‐6 also triggers insulin production in pancreas, hepatic glucose production, and adipose tissue lipolysis, thus ensuring the availability of energy substrates for exercising muscle 11, 17. Moreover, IL‐6 has the ability to induce an anti‐inflammatory response by reducing tumor necrosis factor‐α (TNF‐α) production while increasing the levels of interleukin 10 (IL‐10), interleukin 1 receptor antagonist (IL‐1Ra), and interleukin 15 (IL‐15) 17, 114. Future studies using tissue‐specific IL‐6 knockout mice will be required, however, to fully elucidate to what extent muscle IL‐6 contributes to the positive effects of exercise and to determine whether the anti‐inflammatory effects of IL‐6 are muscle–cell autonomous or not 11, 114.

Irisin, a cleavage product of the protein encoded by the FNDC5 gene, is an exercise‐dependent myokine that was first described in 2012 by Bostrom and colleagues 115. Irisin was shown to trigger WAT remodeling by increasing p38 signaling, UCP1 expression, and therefore browning 115, 116. Irisin can also increase muscle oxidative metabolism and substrate (glucose and fatty acid) uptake, and reduce hepatic glucose output 117. Interestingly, cold‐induced muscle shivering can increase circulating irisin levels and promote brown fat thermogenesis 118 (Fig 3). Moreover, irisin of central (hippocampal) and peripheral origin has been recently shown to promote an increase in cognitive function 119, further highlighting the potential benefits of exercise on healthspan.

Some groups have suggested that irisin can also be produced and secreted by adipose tissue and liver 120. Nevertheless, some inconsistencies have been found between human and mice and in the methodology used 121, which adds more complexity to the functions of irisin in energy homeostasis. In addition, FGF21 has also been proposed to be produced by skeletal muscle in response to the PI3K‐AKT pathway and to act in an autocrine manner to reduce muscle insulin resistance. Moreover, under cold exposure, when shivering thermogenesis is induced, FGF21 can synergize with irisin to promote BAT non‐shivering thermogenesis 118.

Finally, a recent study using proteomic approaches demonstrates that the secreted form of lysosomal enzyme cathepsin B (CTSB) is also an exercise–responsive myokine that targets the brain, promotes hippocampal neurogenesis, and therefore enhances cognitive abilities 122.

Overall in this section, we described how, in order to satisfy the energy demands generated by exercise, muscle communicates with other organs to ensure energy substrate availability. Moreover, physical exercise also promotes the browning of WAT and BAT thermogenesis, possibly in order to meet the increased demand for fatty acid and glucose oxidation and to prevent the accumulation of energy substrates in the circulation 123, 124. Importantly, during exercise, muscle can signal directly to the brain to promote cognitive function, providing an additional support for lifestyle modifications including an increase in physical activity 125.

## Inter‐organ communication in pathological conditions: the case of obesity and related disorders

In pathological situations, WAT can account for more than 50% of human body weight. The increasing prevalence of obesity has triggered considerable interest in understanding the physiological mechanisms promoting calorie storage, and energy expenditure, in order to prevent the deleterious metabolic consequences of obesity such as hyperglycemia, insulin resistance, dyslipidemia, and hepatic steatosis. Insulin resistance is characterized by a decrease in insulin‐stimulated glucose uptake in adipocytes and muscle cells, as well as by a blunted insulin‐stimulated repression of hepatic glucose production 126. Most humans with obesity, as well as DIO mice, fed highly caloric palatable diets, develop insulin resistance, and exhibit high plasma levels of leptin 35. Despite this, they do not show the expected reduction in food intake and increase in energy expenditure, thus leading to the concept of leptin resistance, probably due to a decrease in JAK2‐STAT3 signaling in pathologic conditions 127. In obesity conditions, pathological leptin signaling also contributes to sustained hepatic glucose production, due to abnormal activation of ARC neurons 128. Obesity is also accompanied by an increased NPY expression in the ARC. This increase triggers a reduction in the expression of tyrosine hydroxylase neurons in the paraventricular nucleus (PVN) of the hypothalamus and consequently, diminishes the thermogenic activation of BAT and overall energy expenditure 129. Circulating asprosin levels are higher in obese humans and mice 60. Due to the orexigenic effects of this hormone, this alteration could contribute to the difficulties to obtain a voluntary reduction in food intake in obese patients 60.

Alternatively, mouse models of obesity and unhealthy obese individuals exhibit lower circulating levels of adiponectin. This finding that adiponectin levels are proportional to metabolic fitness has prompted the use of recombinant adiponectin and other adiponectin receptor agonists in several mouse models of obesity. So far, the activation of adiponectin signaling has improved glucose tolerance, insulin sensitivity, and longevity in mice 12, 130. Importantly, adiponectin has been shown to improve the metabolic profile in leptin deficient ob/ob mice by overall increasing energy expenditure, despite not triggering a reduction in food intake 131. In pathological conditions of obesity, the reduction in CSF levels of adiponectin that is accompanied by feeding is lost, thus maintaining a “high food intake behavior” even in conditions that are not of energy scarcity (high energy conditions) 56. As mentioned above, the levels of the gluconeogenic adipokine asprosin are elevated in obese mice. This observation suggests that high plasma asprosin may contribute to the onset of hyperglycemia and insulin resistance in mice and humans. Moreover, the improvement of the metabolic phenotype of obese insulin‐resistant mice when using an antibody to sequester circulating asprosin makes this novel adipokine a potential therapeutic target against diabetes 59.

Interestingly, some studies suggest that the beneficial effects of FGF21 in glucose homeostasis are at least partially mediated by an increase in adiponectin production by WAT 132. However, other groups show that adiponectin is dispensable for the metabolic effects of FGF21 133. Moreover, despite evidence showing improved metabolic fitness in FGF21 treatment or overexpression studies, clinical studies in humans have revealed high FGF21 circulating concentrations in obese and insulin‐resistant subjects, as well as the onset of a so called “FGF21 resistance” 134. These findings highlight the fact that the importance of FGF21 in metabolic health is still debated.

An example of the use of novel strategies for the discovery of endocrine interactions with physiological relevance for whole‐body metabolism is the recent discovery of a novel adipokine, mouse lipocalin 5 (Lcn5; LCN6 in humans), that has the ability to promote insulin sensitivity by enhancing muscle mitochondrial function 135. Seldin and colleagues developed a bioinformatic framework that takes advantage of publicly available data generated with different OMICS approaches to identify new circuits of inter‐organ communication, thus contributing to our understanding of the mechanism by which WAT communicates with other organs to maintain homeostasis. Of note, despite showing significant effects in promoting muscle mitochondrial function in cellular models, the effects of the novel adipokine Lcn5 in promoting glucose and insulin sensitivity *in vivo* were more pronounced in a high‐fat high‐sucrose (HF‐HS) model. However, it is worth noting that Lcn5 overexpression had positive effects on the metabolic status whether at the beginning or after several weeks of HF‐HS diet 135. The use of unbiased genetic and proteomic approaches also allowed the identification of a novel secreted protein, Meteorin‐like (Metrnl), that can be produced by exercising muscle and cold exposed adipose tissue. Metrnl promotes adipose tissue browning by recruiting IL‐4 and IL‐13 producing eosinophils and thus promoting anti‐inflammatory macrophage activation 136. However, serum Metrnl levels are decreased in patients with T2D and inversely correlate with fasting glucose levels 137. These findings highlight the fact that improving muscle oxidative capacity is beneficial both to prevent metabolic disease and to cure metabolic disease; however, the role of Lcn5 and Metrnl in normal physiology remains to be determined.

The effect of gut hormones is also altered in obesity. Indeed, ghrelin has lower orexigenic activity in DIO rodents due to a reduction in activation and plasticity of NPY/AgRP neurons 138. Moreover, a reduced incretin effect is seen in subjects with type 2 diabetes (T2D) and obesity. Interestingly, postprandial glucagon‐like peptide‐1 (GLP‐1) secretion is increased in obese patients following bariatric surgery and correlates with weight loss maintenance 139.

Despite the positive effects of muscle‐derived IL‐6 that are mentioned in the previous section, deleterious IL‐6 can also be released by adipose tissue. Chronically, high levels of circulating IL‐6 in response to HFD contribute to obesity by promoting macrophage recruitment to WAT 140. In conditions of insulin resistance, WAT secretome is substantially altered and includes a broad range of proinflammatory factors, such as TNF‐α, IL‐6, interleukin 8 (IL‐8), and interleukin 1β (IL‐1B) and monocyte chemotactic protein 1 (MCP1) 12. The secretion of these proinflammatory cytokines and chemokines, as well as the secretion of other adipokines like retinol binding protein 4 (RBP4) 141, 142, is now well admitted to contribute to the onset of the physiological alterations accompanying obesity 12.

Physical inactivity, that can be both a cause and a consequence of obesity, is known to be associated with decreased insulin sensitivity, reduced postprandial lipid metabolism, decreased muscle mass, and obesity. Alterations in myokine production associated with physical inactivity may also mediate the onset of obesity. This may be the case for myostatin (also known as GDF8), a member of the TGF‐β superfamily. Myostatin was first described to be secreted during development to limit muscle growth 143, but is also known today to be produced in adults 144, and to promote muscle atrophy, in part by inhibiting AKT and mTOR signaling. Importantly, the inhibition of myostatin signaling increases PGC1α and mitochondrial biogenesis 145, while mice deficient for myostatin show an improved metabolic phenotype and a resistance to HFD 146, 147. However, myostatin‐deficient mice also develop insulin resistance via alterations in AMPK activity 148, highlighting the need to determine the exact effects of myostatin signaling, as well as of other myokines correlating with obesity, in whole‐body energy homeostasis 112.

Overall, the finding that inter‐organ communication is altered in conditions of obesity (Table 1) has gathered increasing attention for researchers aiming to manipulate those preestablished axes to counteract metabolic alterations.

**Table 1:** Alterations of inter‐organ communication in obesity

Molecule	Situation in pathophysiological conditions	References
Leptin	Leptin resistance in DIO mice or obese humans	127
NPY	NPY expression is increased in obesity and leads to decreased BAT thermogenesis and increased food intake	129
Adiponectin	Adiponectin secretion is diminished in obese mice and unhealthy obese humans, thus impairing glucose tolerance and insulin sensitivity	12 130 131
FGF21	Increased FGF21 levels are found in obese individuals, even though an increase in FGF21 is usually associated with metabolic fitness	134 153
Asprosin	Obese mice and humans have increased asprosin levels, associated with hyperglycemia, insulin resistance and increased appetite	60
Lcn5	Increasing Lcn5 (LCN6 in humans) levels promotes insulin sensitivity by enhancing muscle function in murine DIO models	135
Metrnl	Circulating Meteorin‐like levels are significantly lower in T2D patients. Serum Metrnl is inversely correlated with fasting glucose in humans. Accordingly, in mice, Metrnl has been found to stimulate energy expenditure and WAT browning	136 137
Ghrelin	In DIO mice, increased levels of ghrelin do not reduce food intake, due to an impaired downstream activation of neurons. This is defined as ghrelin resistance	138
GLP‐1	GLP‐1 secretion is higher after bariatric surgery and is associated with the important decrease in body weight that accompanies this intervention	139
IL‐6	The high levels of circulating IL‐6 found after HFD feeding in mice and in obese subjects promote inflammatory response in WAT	12 140
RBP4	RBP4 is increased in metabolically unhealthy obese and contributes to the physiological alterations of obesity, such as insulin resistance and dyslipidemia	141 142
Myostatin	Inhibiting myostatin signaling increases mitochondrial biogenesis but also insulin resistance	146 147 148

## Taking advantage of inter‐organ communication to treat obesity and associated diseases

Understanding the biological networks that are involved in the induction of energy expenditure and promote satiety is crucial to target metabolic organs to increase weight loss in the context of the current obesity epidemic. Therefore, numerous efforts are currently being made to further understand the role of the signaling molecules described in this review and to identify novel mechanisms of inter‐organ communication that could contribute to the establishment of new therapeutic approaches against obesity and diabetes. Efforts to include recombinant adipokines or adipokine analogs as pharmacotherapy against obesity began more than 20 years ago. However, most of these efforts have not yet obtained significant success. Indeed, leptin therapy alone has failed to induce significant weight loss or insulin sensitization 149, 150. However, the use of leptin analogs in combination with other agents, namely amylin, has given more encouraging results, but was stopped due to adverse secondary effects 12. Importantly, despite apparently promoting hepatic glucose output by increasing the availability of gluconeogenic substrates in the presence of insulin, in conditions in which insulin levels are low, like in T2D, leptin inhibits lipolysis thus decreasing the circulating levels of gluconeogenic substrates such as glycerol, fatty acids, and ketone bodies, which may play a role in suppressing gluconeogenesis under leptin therapy 41. Adiponectin receptor analogs have successfully been used in preclinical mouse models 130, but have not yet been used in human patients. A truncated globular form of adiponectin has also been shown to promote muscle fatty acid oxidation, decrease muscle ceramides, and overall improve insulin sensitivity, whether or not this globular adiponectin mimics the function of the multimeric adiponectin complexes found in circulation remains to be demonstrated 39. More recently, a neutralizing antibody against asprosin was used in obese mice to reduce appetite, body weight, and blood glucose, suggesting that this orexigenic hormone can be targeted to modulate food intake and lower glycemia in obese subjects 60.

Due to the potential therapeutic effect of increasing overall energy consumption by promoting brite and brown adipose cell metabolism, recent research has focused on the identification of natural hormones or compounds with the capability to promote thermogenic function, such as FGF21, irisin, and BAs 151. Extensive efforts have also been made with the use of recombinant FGF21 or FGF21 variants as potential therapy, due to beneficial metabolic effects observed in mice 152, 153; however, only a small trend toward glucose lowering was reported in humans 154. Moreover, there is increased concern about the use of FGF21 in therapy due to potential adverse effects on the skeletal system, more specifically in bones 155, 156. Finally, despite evidence that irisin treatment can positively affect glucose and lipid metabolism in mice, no real consensus exists as of yet concerning the link between irisin and the metabolic syndrome in humans, and no irisin analog has been used for studies in humans 117.

Significant preclinical and clinical success, however, has been encountered by using GLP‐1 receptor agonists (GLP‐1Ras), that trigger a reduction in body weight by promoting satiety and reducing food intake, and profit from a longer half‐life than endogenous GLP‐1. Moreover, GLP‐1Ras improve glycemic control and reduce liver inflammation and fibrosis 157. More recently, GLP‐1 and glucagon receptor co‐agonists have been successfully used in preclinical studies for metabolic diseases 158, but additional research is necessary to demonstrate their efficacy in humans.

As of now, most potential therapies that take advantage of inter‐organ communication for the treatment of metabolic syndromes remain based on lifestyle alterations: increased physical activity and decreased energy intake.

## Concluding remarks

There is still much work to be done to understand the complex inter‐organ networks that are needed to coordinate energy homeostasis. Recent studies show that more than 15% of the protein‐coding genome encodes for roughly 3,000 secreted proteins, but only a handful of them has been properly annotated 159, 160, suggesting that a very high number of “molecular messengers” remain to be discovered. More recently, population‐based methods taking advantage of the natural variations between transcript levels within strains of a mouse reference population have been used to identify new inter‐organ communication networks in metabolism 135. This bioinformatic‐based approach allowed for the identification of Lcn5, an adipokine that promotes muscle mitochondrial function, and of Notum, a hepatokine that promotes WAT browning and BAT thermogenesis 135. Comparing RNA sequencing of jejunum and stomach when comparing sham and gastric sleeve bypass allowed the identification of LEAP2, an endogenous antagonist of the ghrelin receptor, which may contribute to the efficiency of gastric bypass 47. A similar approach, based on a transcriptomics data mining strategy, was used to identify CXCL14, a novel batokine that promotes thermogenesis by facilitating the recruitment of anti‐inflammatory macrophages to BAT 161.

Moreover, the analysis of large datasets generated with metabolomics and lipidomics studies has shown that different kinds of metabolites, like lipids, amino acids, ketone bodies, and BAs can directly modulate cellular metabolic responses not only by acting as substrates for metabolic reactions, but also as signaling molecules by directly activating different signaling pathways via specific membrane receptors 162. Two recent elegant examples of the importance of metabolites in maintaining energy homeostasis via inter‐organ communication were published < 2 years ago. In one instance, Simcox and colleagues showed that upon cold exposure, WAT produces free fatty acids that, in turn, promote hepatic acylcarnitine production. These acylcarnitines are *in fine* used by BAT for adaptive thermogenesis 163. In addition, Mills and colleagues demonstrated that succinate accumulates in BAT upon cold exposure and favors thermogenesis in an UCP1‐dependent manner 164.

Furthermore, gut microbiota has been described in the past two decades as a very important player in the regulation of whole‐body energy homeostasis, in response to diverse stimuli such as cold 165, 166, nutrition 167, 168, or fasting/feeding cycles 168, 169, 170, through the production of mediators such as short chain fatty acids (SCFAs) and secondary BAs 167, 169.

Finally, vesicles enabling the transfer of molecules from one tissue to the other may regulate systemic metabolism. Exosomes are such vesicles. More precisely, adipose tissue exosomes carrying circulating miRNAs have recently been shown to have far‐reaching effects in other peripheral tissues, and can therefore be considered the most recent family of adipokines 171. For instance, BAT‐produced mir‐99b loaded exosomes contribute to the repression of hepatic FGF21 expression 171.

Overall, the findings summarized in this review highlight the importance of inter‐organ communication in health and disease. They further suggest that multiple stimuli modifying the expression/secretion, the function, and the targets for potentially thousands of molecules (in a broad sense) that affect whole‐body energy metabolism remain to be described/identified.

**Box 1: In need of answers**

In the last decades, increasing efforts have been made to understand the mechanisms behind tissue communication. Omics approaches have been used to characterize novel molecules participating in inter‐organ communication, like Lcn5 and Notum 135, but more research is necessary to understand their role in normal physiology. These novel techniques will allow for the identification of novel players in inter‐organ communication, but identifying their receptors/transporters, understanding their mechanism of action, and defining their target cell type(s) will captivate researchers in the field for the years to come.

Additionally, the study of inter‐organ interactions in different physiological conditions is of high relevance for the optimization of drug efficiency. Indeed, it is well known that fasting/feeding cycles can influence drug efficiency 172; however, the mechanism behind this observation remains mostly unknown. Diet composition, nutrient availability and metabolic status can also modulate pharmacological treatments. Therefore, the direct use of specific diets, or the modulation of inter‐organ communication, could have a pharmacological effect on its own 167 or increase the efficiency of pharmacological approaches to treat a wide variety of diseases 173, 174.

Special efforts must be made to characterize the role of metabolites and lipids as signaling molecules that can potentially act in an autocrine, a paracrine, or an endocrine manner 162. We also believe that special attention should be placed on exosomes. These small, naturally existing vesicles are the perfect vehicle to transfer molecules from cell to cell and tissue to tissue.
